# *In situ* Immune Signatures and Microbial Load at the Nasopharyngeal Interface in Children With Acute Respiratory Infection

**DOI:** 10.3389/fmicb.2018.02475

**Published:** 2018-11-09

**Authors:** Kiyoshi F. Fukutani, Cristiana M. Nascimento-Carvalho, Maiara L. Bouzas, Juliana R. Oliveira, Aldina Barral, Tim Dierckx, Ricardo Khouri, Helder I. Nakaya, Bruno B. Andrade, Johan Van Weyenbergh, Camila I. de Oliveira

**Affiliations:** ^1^Instituto Gonçalo Moniz-FIOCRUZ, Salvador, Brazil; ^2^School of Medicine, Federal University of Bahia, Salvador, Brazil; ^3^Department of Pediatrics, School of Medicine, Federal University of Bahia, Salvador, Brazil; ^4^Department of Microbiology and Immunology, Rega Institute for Medical Research, KU Leuven, Leuven, Belgium; ^5^Department of Clinical and Toxicological Analyses, School of Pharmaceutical Sciences, University of São Paulo, São Paulo, Brazil; ^6^Multinational Organization Network Sponsoring Translational and Epidemiological Research Initiative, Fundação José Silveira, Salvador, Brazil

**Keywords:** network analysis, ARI, immune response, nCounter, interferon, viral load, innate immunity, adaptive immunity

## Abstract

Acute respiratory infection (ARI) is the most frequent cause for hospitalization in infants and young children. Using multiplexed nCounter technology to digitally quantify 600 human mRNAs in parallel with 14 virus- and 5 bacterium-specific RNAs, we characterized viral and bacterial presence in nasopharyngeal aspirates (NPA) of 58 children with ARI and determined the corresponding *in situ* immune profiles. NPA contained different groups of organisms and these were classified into bacterial (*n* = 27), viral (*n* = 5), codetection [containing both viral and bacterial transcripts (*n* = 21), or indeterminate intermediate where microbial load is below threshold (*n* = 5)]. We then identified differentially expressed immune transcripts (DEITs) comparing NPAs from symptomatic children vs. healthy controls, and comparing children presenting NPAs with detectable microbial load vs. indeterminate. We observed a strong innate immune response in NPAs, due to the presence of evolutionarily conserved type I Interferon (IFN)-stimulated genes (ISG), which was correlated with total bacterial and/or viral load. In comparison with indeterminate NPAs, adaptive immunity transcripts discriminated among viral, bacterial, and codetected microbial profiles. In viral NPAs, B cell transcripts were significantly enriched among DEITs, while only type III IFN was correlated with viral load. In bacterial NPAs, myeloid cells and coinhibitory transcripts were enriched and significantly correlated with bacterial load. In conclusion, digital nCounter transcriptomics provide a microbial and immunological *in situ* “snapshot” of the nasopharyngeal interface in children with ARI. This enabled discrimination among viral, bacterial, codetection, and indeterminate transcripts in the samples using non-invasive sampling.

## Introduction

Acute respiratory infections (ARIs) cause great morbidity and mortality in children across the globe. The ARI is mainly associated with viral infections and it is generally confined to the upper respiratory tract, causing cough and hoarseness (Tregoning and Schwarze, 2010). However, it may extend to the lower respiratory tract, which in turn causes wheezing and respiratory distress. Concomitant viral and bacterial infection, caused mainly by *Streptococcus pneumoniae, Haemophilus influenzae*, and *Moraxella catarrhalis*, can lead to the development of acute otitis media (Ruohola et al., 2013) or pneumonia (Nolan et al., 2017). This adds another layer of complexity to the microbial interactions at the respiratory tract.

Transcriptome analyses provide a comprehensive and efficient way to elucidate the host immune response to infectious diseases (Chaussabel et al., 2010; Nakaya et al., 2012) and vaccines (Nakaya et al., 2016). Recent investigations have used blood transcriptomic analysis to identify immune signatures in community-acquired pneumonia (CAP) (Parnell et al., 2012), respiratory syncytial virus (RSV) infection (Mejias et al., 2013), and rhinovirus (RV) infection (Heinonen et al., 2015). Additionally, such analyses are robust enough to distinguish bacterial from viral respiratory infection, suggesting that transcriptional profiling can improve diagnosis (Suarez et al., 2015; Sweeney et al., 2016). In parallel, studies performed at the nasopharynx interface have shed light on the relationships between different microorganisms that may colonize this site such as *S. pneumoniae, H. influenzae*, and those that can cause infection (RSV and RV) (Wiertsema et al., 2011; Skevaki et al., 2015; Uitti et al., 2015).

We have previously reported the use of nCounter technology to identify viral and bacterial agents in nasopharyngeal aspirates (NPAs) of children with ARI, enrolled at a reference pediatric emergency unit in Brazil (Fukutani et al., 2015). In the present study we determined microbial (viral and bacterial) loads and performed immune transcriptional profiling in NPAs of those children with ARI. Systems immunology approaches identified modular communities of immune response genes that are correlated with microbial load, thus distinguishing microbe-related immune signatures.

## Materials and Methods

### Ethics Statement

All legal guardians of pediatric patients and adult participants signed a written term of informed consent. This study complied with the guidelines of the Declaration of Helsinki and was approved by the Institutional Review Board of the Federal University of Bahia (protocol no. 067/2009).

### Study Design and Patients

We conducted this prospective cross-sectional study at the pediatric emergency room of the Federal University of Bahia hospital, in Salvador, northeastern Brazil. Recruitment of children (aged 6 to 23 months) presenting with symptoms consistent with ARI (*n* = 576) occurred from September 2009 to October 2013 (Bouzas et al., 2016). The RNA extraction was performed in a randomized subset of NPAs (*n* = 58), which corresponded to 10% of the total cohort of children with ARI (*n* = 576). Inclusion criteria consisted of reports of fever, sneezing, rhinorrhea, nasal blockage, or coughing for up to 7 days. Exclusion criteria were transfer from another hospital or a reported previous episode of wheezing. Pediatricians performed physical examinations and collected data using a standardized form. A sample of healthy controls (*n* = 7, adults) was recruited by convenience, with inclusion criteria consisting of the absence of fever, cough, sore throat, sneezing, nasal blockage, or rhinorrhea for 14 days prior, in the absence of nasal medication usage.

### Collection of Nasopharyngeal Aspirates and RNA Extraction

Nasopharyngeal aspirate samples were collected from each enrolled subject. Briefly, the distance between the entrance of the nostril and the ear lobe was used as an estimate of the distance from the entrance of the nostril to the nasopharynx. An aseptic catheter was inserted into the nostril and upon reaching the nasopharynx, negative pressure was applied to collect approximately 2 mL of nasal secretion. Samples were placed in a sterile tube containing 1 mL of NucliSENS Lysis Buffer (bioMerieux) and frozen at -70°C until use. For healthy controls (*n* = 7), the collection of NPAs was performed by instilling 2 mL of isotonic saline into each nostril, followed by immediate aspiration (∼1 mL) via the insertion of a flexible catheter. These samples were transferred into sterile tubes containing 1 mL of NucliSENS Lysis Buffer (bioMerieux) and frozen at -70°C until use. Total ribonucleic acid (RNA) was obtained using an RNEasy Kit (Qiagen) in accordance with manufacturer’s instructions. The RNA was quantified using a Qubit RNA HS Assay (Invitrogen) and hybridized (50 ng) against custom-designed probes using nCounter to detect the following: adenovirus (AV) 2 and 5, human bocavirus (hBoV), coronavirus (CoV) 229E and OC43, RSV A and B, influenza virus (IV) A and B, parainfluenza virus (PIV) 1, 2, and 3, RV, human metapneumovirus (hMPV), *Chlamydia pneumoniae, H. influenzae, M. catarrhalis, Mycoplasma pneumoniae, and S. pneumoniae* (Fukutani et al., 2015). The RNA was also hybridized against immune targets using the immunology custom set V2^1^. All the probes were synthesized by NanoString Technologies^®^ and nCounter (NanoString^®^) reactions performed at the Nucleomics Core Facility (VIB, Leuven, Belgium), as previously described (Geiss et al., 2008).

### NPA Microbial Load Determination

As described before in Fukutani et al. (2015) and Bouzas et al. (2018), the nCounter probe set used in this study was extensively tested and optimized for semihigh throughput analysis. First, sensitivity, linearity, and absence of cross-reaction among the respiratory bacterial and viral targets were tested with a panel of positive controls obtained from the collection of the Laboratory of Clinical Virology (KU Leuven). Second, we performed a pilot experiment in a randomized subset of samples from the cohort (*n* = 58). Due to financial limitations, only the pathogens reliably detected in both stages were used in subsequent experiments. Nonetheless, we have confirmed the detection of RSV-A/RSV-B, as shown here by nCounter, using RT-PCR (Bouzas et al., 2018), and of *H. influenzae, M. catarrhalis, S. aureus*, and *S. pneumoniae* by 16S next-generation sequencing (Andrade et al., 2016).

Raw data were preprocessed using both nSolver 2.0 software (NanoString Technologies^®^) and the NanoStringNorm R package (Waggott et al., 2012), as described by Fukutani et al. (2015). In this standardized protocol, we sequentially corrected two factors for each individual sample: technical variation between samples (using spiked positive control RNA) and background correction (using spiked negative control RNA). This method allows the identification of multiple pathogens in NPA samples of ARI patients in a single reaction, which present low RNA yield (10–50 ng) (Fukutani et al., 2015). Pathogen transcript counts were log10 transformed. Each individual’s microbial load was determined by comparing the number of transcripts to that of the individual’s cutoff value, established as the mean (plus three standard deviations) of eight internal negative control reactions. The NPAs with microbial counts above their respective cutoffs were considered pathogen-positive and NPAs with microbial counts below the calculated cutoff were considered indeterminate (Supplementary Dataset 1). The NPAs considered positive were further classified as “bacterial,” “viral,” or “codetected” (i.e., presenting both viral and bacterial transcripts).

### Data Analysis

To identify relationships between nodes (bacteria and virus present in NPAs), we constructed an association matrix using Gephi, and the network distribution was created using Force Atlas algorithm (Fukutani et al., 2015). Immune response transcripts were quantified as described earlier for microbial pathogens, except for log2 transformation. Differentially expressed immune transcripts (DEITs) were determined by multiple *t*-tests employing Benjamini-Hochberg correction and false discovery rate (FDR) set at 5% (the full list of genes is shown in Supplementary Dataset 2). The DEITs were determined by comparing the transcripts of ARI patients with healthy controls. Pathway enrichment of DEITs was performed using gene set enrichment analysis (GSEA) (Subramanian et al., 2007). The GSEA was run on preranked individual genes (Log2 values of the 600 genes analyzed) using the Reactome database (nominal *p* < 0.05; 1,000 permutations) (Fabregat et al., 2017).

Correlations between DEITs (Spearman, *p* < 0.05) and NPA microbial load were determined using R (R Core Team, 2013) and the correlation strength was assessed by non-parametric bootstrap (100× replicates) and only those with value over 50% were considered. Modularity analysis (Blondel et al., 2008) was performed to identify nodes that are more densely interconnected (Supplementary Dataset 3). The DEITs significantly correlated with microbial load, and those present in >50% in bootstrap resampling were considered as a community, whereas DEITs below this threshold were marked as bystanders. For the sparse partial least squares (sPLS) approach implemented in the R package (Liquet et al., 2016), DEITs were used as ‘predictors’ and the microbial load as ‘response variables.’ This method disintegrates the predictors and the response variation, while identifying their correlation and the contributors for each variation component. The sPLS score was then used in a Spearman correlation analysis against the bacterial or the viral load to confirm the role of correlated DEITs. In addition, DEITs were also determined by comparing NPAs of ARI patients with classified microbial load (“bacterial,” “viral,” or “codetected”) to ARI patients classified “indeterminate.” Cell-specific gene lists validated by CTEn (cell type enrichment) (Shoemaker et al., 2012) were used to identify enriched cell types in the immune signature observed in these comparisons. Correlation and modularity analyses were also performed with respect to these DEITs (Supplementary Datasets 4, 5). To derive an evolutionarily conserved antiviral score, we used a genome-wide cross-species comparison of type I IFN-induced antiviral response and tallied the number of species (0–10) with significant (FDR < 0.05) antiviral activity for each individual gene (Shaw et al., 2017).

## Results

### Microbial Load and Clinical Symptoms of Children With ARI

**FIGURE 1:**
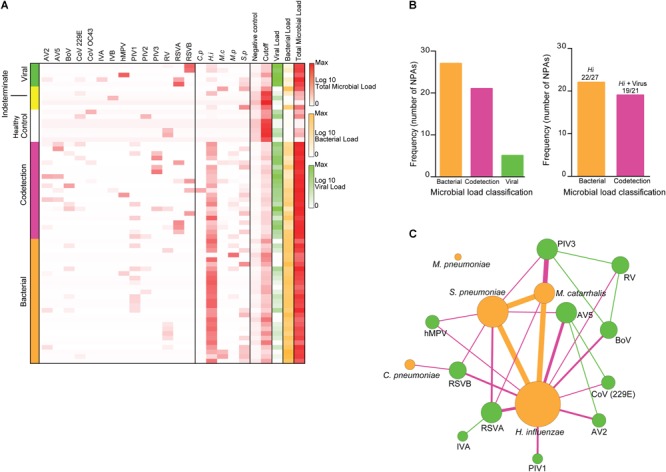
Microbial composition of nasopharyngeal aspirates of children with ARI. Nasopharyngeal aspirates (NPAs) (*n* = 53) were subjected to transcriptomics analysis using a set of probes targeting adenovirus (AV 2 and 5), bocavirus (BoV), coronavirus (CoV 229E and OC43), parainfluenza virus (PIV 1, 2, and 3), respiratory syncytial virus (RSVA and B), influenza virus (IV A and B), human metapneumovirus (hMPV), and rhinovirus (RV), *Haemophilus influenzae, Streptococcus pneumoniae, Mycoplasma pneumoniae, Moraxella catarrhalis*, and *Chlamydia pneumoniae.* **(A)** Heatmap depicting the number of pathogen transcripts (Log10) for each NPA; NPAs were classified according to the microbial load: Viral (green), Codetection [presence of both viral and bacterial transcripts) (pink)], and bacterial (orange). Control NPAs were obtained from healthy subjects (See Materials and Methods and Supplementary Dataset 1 for details). **(B)** Frequency of NPAs classified according to the microbial load classification (left). Frequency of NPAs presenting *H. influenzae* transcripts in NPAs classified as bacterial or codetection. **(C)** Association network depicting associations among the different microbial targets detected in NPAs. Size of circle indicates node degree and line width indicates correlation strength.

In this prospective study of 58 randomized NPA samples (corresponding to 10% of the complete cohort, *n* = 576) from children diagnosed with ARI, we initially determined the microbial load of the NPAs using custom-designed probes and nCounter digital transcriptomics. Of the 58 NPAs evaluated, 53 (91.4%) samples showed microbial transcript counts above the calculated cutoffs and were considered positive. These NPAs were further classified as “bacterial” (*n* = 27, 46.6%), “viral” (*n* = 5, 8.6%), or “codetection” (presenting both bacterial and viral transcripts) (*n* = 21, 36.2%) (Figure 1A and Supplementary Dataset 1). Five NPAs (8.6%) were classified as “indeterminate,” because their microbial transcript counts were below the calculated cutoff, and thus displayed a microbial profile similar to healthy controls (Figure 1A and Supplementary Dataset 1). Among the 27 bacterial NPAs, 22 (81.5%) presented with *H. influenzae* transcripts (Figure 1B). Transcripts for *S. pneumoniae* (8/58 NPAs, 13.8%) and *M. catarrhalis* (2/58 NPAs, 3.4%) and for *M. pneumoniae* and *C. pneumoniae* (1/58 NPA each, 1.72%) were also detected (Figure 1A and Supplementary Dataset 1). In viral NPAs, we detected transcripts for RSVA (1/58, 1.7%), RSVA + IVA (1/58, 1.7%), hMPV (1/58, 1.7%), and RSVB (2/58, 3.4%) (Figure 1A and Supplementary Dataset 1). Transcripts for CoV, IVB, and PIV2 were not detected in any of the 53 NPAs evaluated. In codetection NPAs, *H. influenzae* transcripts were also overtly represented (19/21 NPAs, 90.5%) (Figures 1A,B and Supplementary Dataset 1) and occurred in combination with transcripts for RSVA, RSVB, PIV1, PIV3, AV2, AV5, hMPV, and CoV229. Upon examination of correlations among the different microbial targets in NPAs (Figure 1C), *H. influenzae* is at the center of the network, indicating that it is associated with all other targets detected, particularly *S. pneumoniae* and *M. catarrhalis.* Viral targets associate mostly with a bacterial target and rarely with another viral target (for e.g., RV and BoV or PIV3).

The baseline demographic and clinical characteristics of the study group are described in Table 1. The mean age of patients with ARI was 11.7 months [(34/53 (58%) females]. The most frequent complaints were coryza (87.9%), cough (86.2%), and sneezing (84.4%). Upon physical examination, the most frequent findings were fever (82.7%) and wheezing (37.9%). Notably, we did not observe significant differences in either verbal complaints or any findings following physical examination of children harboring NPAs classified as bacterial, viral, or codetection (Table 1). Therefore, in this cohort, ARI symptoms could not be associated with specific respiratory pathogens, nor with pathogen classes (viral vs. bacterial). These data are not shown. Of note, strict viral presence was observed in only 5/58 (8.6%) NPAs, while *S. aureus* transcripts were detected in similar numbers across all NPAs examined and, hence, were not included in our analysis of pathogen-related immune signatures (Supplementary Figure 1).

**Table 1:** Demographic and clinical characteristics of children with ARI (*n* = 58).

Symptoms	Frequency^∗^
Age (months)	11.7 (6.1–23.6)
Gender (F:M)	34:24
Temperature (°C)	38.5 (37.5–40.0)
Coryza	51 (87.9%)
Cough	50 (86.2%)
Fever	48 (82.7%)
Sneezing	49 (84.4%)
Nasal Occlusion	47 (81.0%)
Wheezing	22 (37.9%)
Hoarseness	19 (32.7%)
Dyspnea	16 (27.5%)
Vomit	12 (20.6%)
Otalgia	6 (10.4%)
Cyanosis	2 (3.4%)
Diarrhea	2 (3.4%)

*^∗^Frequency is shown as median values (min–max range) or number (percent).*

### Immune Signature at the Nasopharyngeal Interface of Children With ARI

**FIGURE 2:**
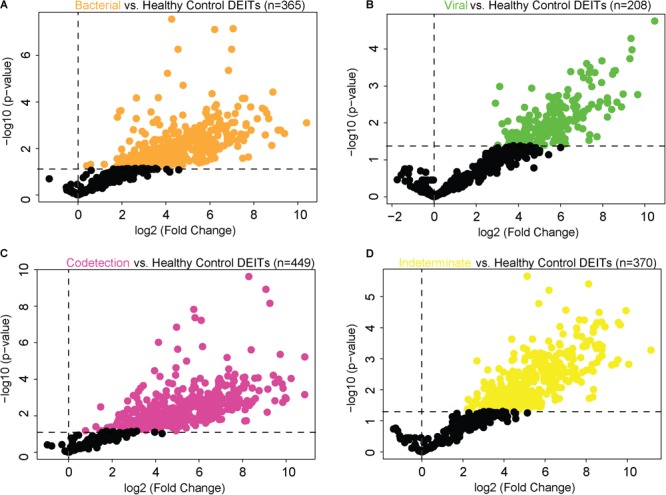
Differentially expressed immune genes in nasopharyngeal aspirates of children with ARI. “Volcano plots” of statistical significance against fold change between NPAs harboring microbial load [classified as bacterial, orange **(A)**; viral, green **(B)**; codetection, pink **(C)**; and indeterminate, yellow **(D)**] and NPAs from healthy controls, demonstrating the number of differentially expressed immune genes.

Following the determination of the microbial load at the nasopharyngeal interface, we comprehensively explored the immune signature, also by transcriptomic profiling, using a large panel of 598 prespecified immune response-related genes. In this initial exploratory analysis, the DEITs were determined by comparing NPAs of children with ARI with those NPAs of healthy controls (Supplementary Figure 2). Bacterial NPAs displayed an upregulation in 365 genes (Figure 2A), whereas 208 genes were upregulated in viral NPAs (Figure 2B). Codetection NPAs showed the highest number of upregulated immune genes (*n* = 449) (Figure 2C). As mentioned previously, five NPAs were classified as “indeterminate,” as the microbial transcript counts were below the established cutoff and indistinguishable from healthy controls, but these samples also displayed upregulated genes (*n* = 370) (Figure 2D). Using GSEA, bacterial NPAs showed the broadest enrichment of pathways whereas viral NPAs were expectedly related to antiviral immunity, interferon and IRF7 activation (Supplementary Figure 3). Bacterial, viral, and codetection NPAs were all enriched for Type I and Type II interferon signaling.

### Identification of Modular Communities of Genes and Correlation With Microbial Load

**FIGURE 3:**
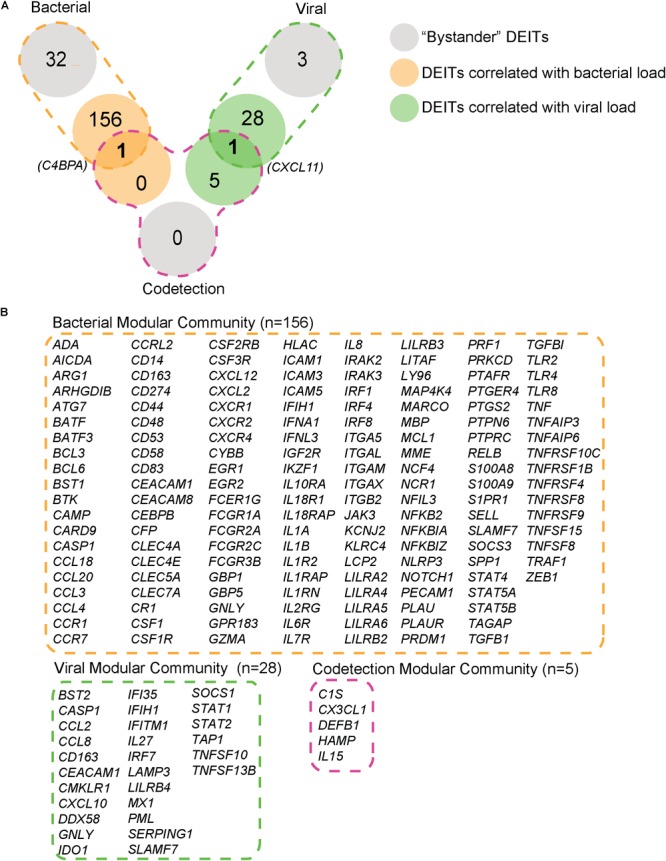
Modules of immune genes differentially expressed in nasopharyngeal aspirates of children with ARI. The correlation between DEITs (differentially expressed immune transcripts) and microbial load in NPAs classified as bacterial (orange) or viral (green) were determined by Spearman (*p* < 0.05) and correlation strength was assessed by non-parametric bootstrap. The DEITs with bootstrap values >50% were subjected to modularity analysis (see Materials and Methods for details). **(A)** Numbers within the orange or green circles represent genes with similar modularity. Numbers within gray circles represent genes with different modularities. **(B)** Modular communities of DEITs correlated with bacterial, viral, or codetection (bacterial + viral) load.

We next used modularity analysis to identify DEITs that are most densely connected together and that correlated with microbial load. Among the 365 DEITs upregulated in bacterial NPAs (shown in Figure 2A), 156 DEITs formed a module/community that was directly correlated with bacterial load (Figure 3A), whereas the remaining 32 genes formed a separate module/community (Supplementary Dataset 3). On the contrary, among the 208 DEITs detected in viral NPAs (shown in Figure 2B), the module/community correlated with viral load consisted of 29 genes (Figure 3A). Codetection NPAs presented the highest number of upregulated genes, but the community/module that correlated with microbial presence contained only five genes (*C1S, CX3CL1, DEFB1, HAMP1*, and *IL15*). Notably, *C4BPA*, a gene that encodes a protein involved in complement activation, was differentially expressed and correlated with bacterial load in bacterial and in codetection NPAs, whereas *CXCL11*, a chemokine that recruits activated T cells, neutrophils, or monocytes, was correlated with microbial load in viral and in codetection NPAs (Figure 3A).

**FIGURE 4:**
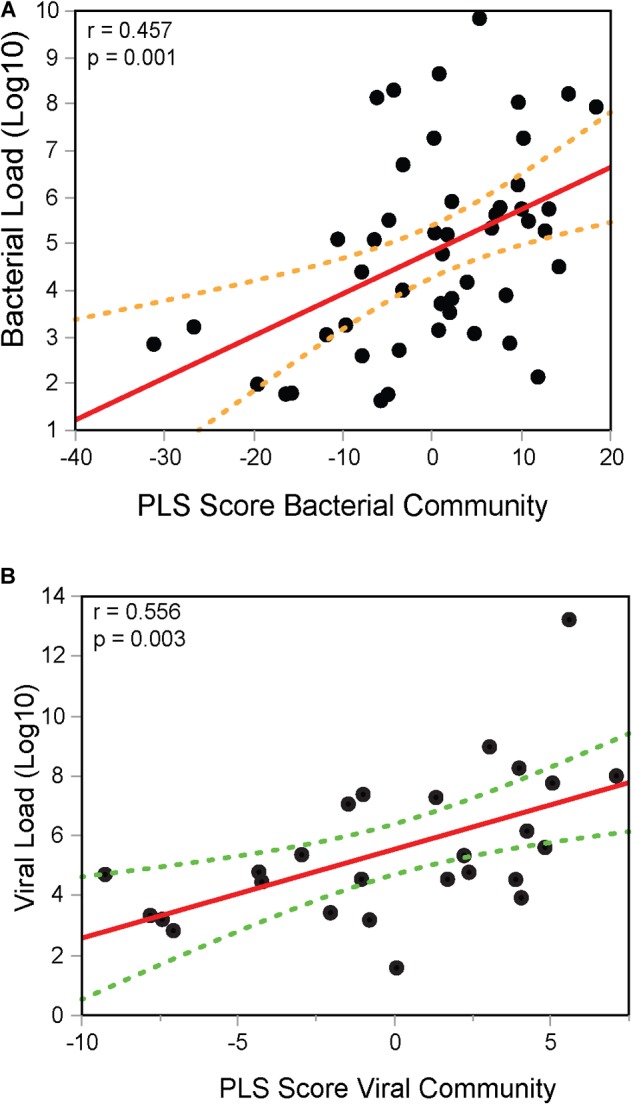
Integrative analysis of gene expression module and microbial load in children with ARI. Bacterial **(A)** or viral **(B)** loads were correlated with partial least square (PLS) scores obtained for the modular communities of DEITs.

Therefore, *in situ* transcriptomics revealed three communities/modules: the first module consisted of 157 (156 depicted in the module plus *C4BPA*) immune genes, whose expression is directly correlated with bacterial counts (Figure 3B), which herein is mostly *H. influenzae* (shown in Figure 1). A second modular community consisted of 29 (28 genes depicted in the module plus *CXCL11*) genes (Figure 3B), whose expression was directly correlated with viral counts (RSV, PIV, AV, etc.). The last community consisted of five genes, whose expression was correlated with both bacterial and viral transcripts (codetection NPAs). We then applied sPLS regression to confirm that the modules/communities of immune genes identified previously were correlated with the microbial load. In this step, bacterial and codetection NPAs were analyzed together, since both presented transcripts for bacteria, as were viral and codetection NPAs, since both presented viral transcripts. Indeed, we observed a strong positive correlation (*r* = 0.457 and *p* = 0.001) (Figure 4A) between the bacterial module of immune genes (shown in Figure 3B) and bacterial load. This result was also obtained (*r* = 0.556 and *p* = 0.003) (Figure 4B) when we compared the viral module of immune genes (shown in Figure 3B). We thus identified an *in situ* gene signature that is specifically attributed to the quantity of bacterial and/or viral transcripts in NPAs of children with ARI.

### Indeterminate NPAs Present a Distinct Immune Signature

**FIGURE 5:**
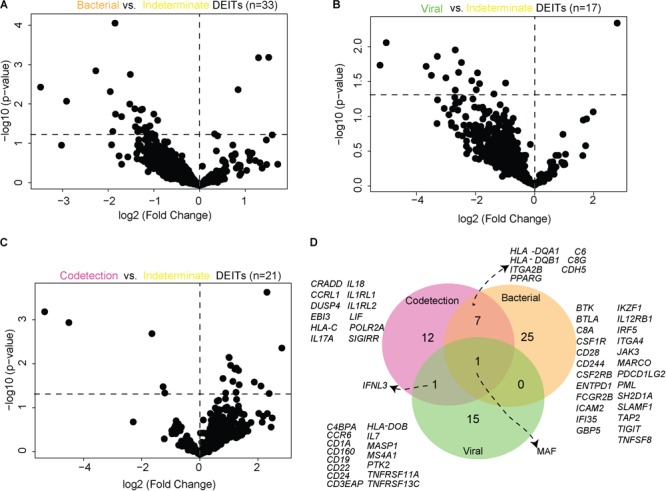
Differentially expressed immune transcripts in nasopharyngeal aspirates of children with symptomatic ARI. “Volcano plots” of statistical significance against fold change between NPAs harboring microbial load [**(A)**, bacterial; **(B)**, viral; and **(C)**, codetection] and NPAs characterized as “Indeterminate,” demonstrating the number of differentially expressed immune genes. **(D)** Venn diagram depicting the differentially expressed genes observed in each comparison.

As shown in Figure 1A, a subset of NPAs was classified as “indeterminate,” since microbial transcript counts were below the established cutoff (similar to healthy controls), despite the clinical symptoms shown by the children. We, therefore, performed a new set of analyses comparing these two groups of NPAs: from children with symptoms and detectable microbial load (viral, bacterial, or codetection) vs. from children with symptoms, but in which microbial load was below the cutoff (indeterminate). We hypothesized that the gene expression profile detected by nCounter could distinguish microbial presence, independent of clinical symptoms. We thus applied the same analysis pipeline (Supplementary Figure 2) to identify whether the immune signatures in bacterial, viral, and codetection NPAs were distinct from those that are present in indeterminate NPAs. Notably, the number of DEITs in bacterial (*n* = 33, Figure 5A), viral (*n* = 17, Figure 5B), or codetection NPAs (*n* = 21, Figure 5C) was lower in comparisons with indeterminate NPAs and in the comparisons with healthy controls (shown in Figure 3). In bacterial and viral NPAs, most of the genes were downregulated (Figures 5A,B, respectively) suggesting that indeterminate NPAs displayed a stronger modulation of the immune response.

Importantly, adaptive immunity transcripts appeared to discriminate viral, bacterial, and codetection NPAs, when compared with indeterminate NPAs. For example, prototypical B cell transcripts (CD19, CD22, CD24, MS4A1, CCR6, HLA-DOB, and BAFF-R/TNFRSF13C) predominated in viral NPAs (Figure 5C). Using CTEn curated lists of cell type-specific genes, we found that B cell transcripts were significantly enriched among viral DEITs (enrichment *p* < 0.0001, OR 20.7 [10.4–39.4]). In bacterial NPAs, myeloid (CSFR1, CSFR2B, MARCO, and IRF5) and coinhibitory transcripts (CD39/ENTPD1, FCGR2B, PD-L2/PDCD1L2, TIGIT, and SAP/SH2D1A) were detected (Figure 5C) and were significantly correlated with bacterial load. According to CTEn classification, monocyte-(BTK, CSF1R, CSF2RB, ENTPD1, ICAM2, IFI35, IKZF1, IRF5, and ITGA4) and myeloid-specific (MARCO) genes were significantly enriched among bacterial DEITs (*p* < 0.0001, OR 29.7 [14.7–61.3]). Strikingly, *IFNL3* was expressed in codetection and in viral NPAs (Figure 5D), in agreement with its unique protective role against respiratory viruses (reviewed in Andreakos et al., 2017). The transcription factor *MAF* was detected in all comparisons suggesting that it is universally expressed in NPAs presenting a detectable microbial load when compared with NPAs presenting with undetectable microbial load (“indeterminate”).

**FIGURE 6:**
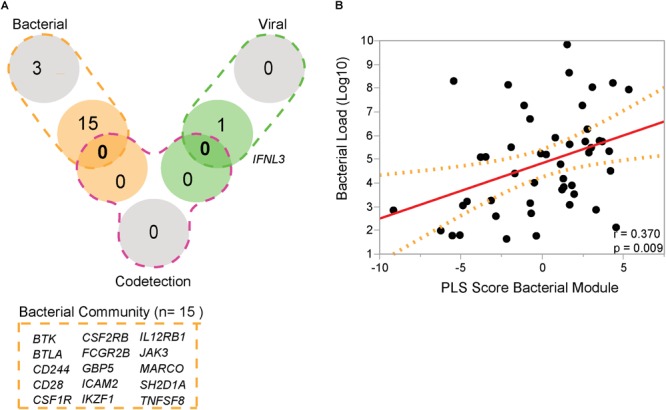
Modules of immune genes differentially expressed in nasopharyngeal aspirates of children with symptomatic ARI. The correlations between DEITs and microbial load in NPAs classified as bacterial (orange) or viral (green) were determined by Spearman (*p* < 0.05) and correlation strength was assessed by non-parametric bootstrap. The DEITs with bootstrap values >50% were subjected to modularity analysis (see Materials and Methods for details). **(A)** Numbers within the orange or green circles represent genes with similar modularity. Numbers within gray circles represent genes with different modularities **(B)** Bacterial load in NPAs classified as bacterial and codetection were correlated with Partial Least Square (PLS) scores obtained for the modular communities of differentially expressed genes depicted in A (orange box).

Similar to the previous comparisons performed with healthy controls (shown in Figure 3), we again identified two modules/communities of immune genes that were associated with either bacterial or viral load (Figure 6A) and, in the latter, the module consisted only of *IFNL3*. The module/community of 15 immune genes associated with bacterial presence was positively correlated with microbial load (*r* = 0.370 and *p* = 0.009) (Figure 6B). These results indicate that bacterial NPAs display an *in situ* immune signature that is specific and also different from that observed in indeterminate NPAs.

**FIGURE 7:**
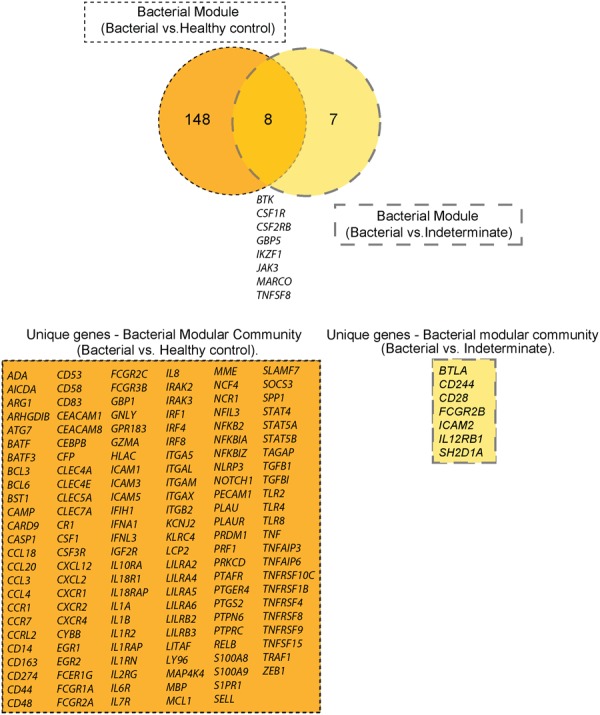
Immune genes differentially expressed in nasopharyngeal aspirates of children with ARI. Venn diagram depicting the DEITs detected in NPAs displaying microbial load or NPAs classified as indeterminate. The intersection shows immune genes common to both categories.

Lastly, we focused on bacterial NPAs and compared the module/community of immune genes detected in the comparisons with healthy control NPAs (shown Figure 3B) vs. those detected in the comparisons with indeterminate NPAs (shown in Figure 6A). We thus identified immune genes that were specifically expressed in children with symptomatic ARI harboring bacterial or viral transcripts at the nasopharyngeal interface (Figure 7). We have also identified another set of immune genes uniquely expressed in symptomatic children with ARI, but in which microbial detection was below the established threshold (“indeterminate”). At the intersection of these two modules, we established a subset of eight immune genes that are expressed in both the conditions, irrespective of the type of microbial presence or the presence of clinical symptoms (Figure 7). This group of genes consisting of bona fide myeloid markers (CSF1R, CSF2RB, and MARCO), costimulatory transcripts (CD28, TNFSF8), but also coinhibitory molecules (BTK, BTLA, FCGR2B, and “SLAM Accessory Protein” SAP/SH2D1A) may represent a general nasopharyngeal immune signature, during ARI.

## Discussion

Despite the global impact of ARI, our understanding of the immune response that occurs at the nasopharyngeal interface is still incomplete. Herein, we characterized the immune response at the NPA interface, in symptomatic children with ARI, displaying microbial loads consisting of bacterial, viral, or a combination of both. The immune response and microbial loads were determined using *in situ* transcriptomics, enabling us to identify modular communities of genes that are expressed in response to bacterial or viral presence.

Nasopharyngeal aspirates were initially characterized and classified according to the microbial transcripts present: the majority of samples harbored either strictly bacterial (47%) or a composition of both bacterial and viral transcripts (codetection) (36%), and a minority displayed the presence of only viral transcripts (<9%). Among bacteria, *H. influenzae* was the most predominant bacterium, which together with *S. pneumoniae* and *M. catarrhalis* makes up the healthy nasopharynx microbiome (Bogaert et al., 2011). Viral presence occurred mostly in presence of bacteria and the most common transcripts were those for RSV A and B, and PIV3. RSV is one of the most common etiological agents of respiratory infection, including pneumonia (Nascimento-Carvalho et al., 2010).

*Haemophilus influenzae* is a component of the healthy upper respiratory tract microbiota (Bogaert et al., 2011), but it can spread causing systemic or localized infection. Colonization of *H. influenzae* begins in infancy and approximately 20% of cases are colonized within the first year of life (Howard et al., 1988). When a new bacterial or viral acquisition occurs, a potential disturbance in the microenvironment can lead to invasion, dissemination, and complications. For example, RV infection stimulated *S. pneumoniae* adhesion to airway epithelial cells (Ishizuka et al., 2003), whereas RSV and PIV2 infection increased the expression of receptors for pathogenic bacteria (Avadhanula et al., 2006). These are two possible mechanistic explanations for complicated disease in simultaneous bacterial/viral infection. We have also shown that pleural effusion, a complication of pneumonia, was shown to occur in children with mixed viral and bacterial presence (Nascimento-Carvalho et al., 2013). Although our analyses confirmed the presence of microbial transcripts in the NPAs, including a number of samples with mixed (codetection) transcripts, clinical symptoms presented by such children did not differ significantly from those harboring a single infection, at least at the time of admission. Regarding viral ARI, increased disease severity in the case of multiple codetection has not been clinically observed (reviewed in Nascimento-Carvalho and Ruuskanen, 2015). However, most of the reports reviewed therein employed DNA detection, which may reflect a range of conditions. This differed from our strategy that employed mRNA detection, thus mirroring active viral and bacterial replication.

Following the classification of NPAs according to their microbial load, we sought to determine the immune signature at the nasopharyngeal interface. To do so, the gene expression of immune response-related genes was compared in NPAs obtained from children with ARI vs. NPAs obtained from healthy controls. Overall, we detected a strong upregulation in gene expression, it being the strongest in NPAs classified as “codetection,” followed by “bacterial” and “viral.” NPA classified as “indeterminate” also displayed clinical symptoms and a significant upregulation in gene expression, which might be explained by two hypotheses that are not mutually exclusive. The first hypothesis is that indeterminate NPAs might contain unknown respiratory or non-respiratory pathogens that were not present in our customized set of probes, which contained all respiratory viruses and bacteria currently considered to be clinically relevant. The second hypothesis is that the “indeterminate” immune signature might be triggered by environmental allergens, in agreement with our recent observation that environmental factors (the presence of dogs and birds in the house) are associated with wheezing in children with ARI (Oliveira et al., submitted). Furthermore, nCounter has proven highly sensitive for the detection of (retro)viral transcripts in HTLV-1-infected T cell lines (Moens et al., 2012), and equally sensitive as real-time PCR for the detection of RSVA and RSVB in NPAs (Bouzas et al., submitted), arguing against a failure of nCounter technology to detect microbial transcripts in indeterminate NPAs.

Functional analysis of the differentially expressed genes pointed to a common pathway enriched across all NPA classification: interferon alpha, beta, and gamma signaling. Type I interferons (alpha/beta) are produced early upon virus infection and signal though the IFNAR receptor to induce genes that encode proteins important for limiting viral replication. Type II interferon (gamma) is produced by lymphocytes and NK cells and is associated with inflammatory responses. Mejias et al. (2013) also found the interferon-signaling pathway to be the most upregulated pathway in blood transcriptomic analysis with a cohort of children with RSV infection. In this study, the authors followed 21 patients with RSV infection and found greater activation of interferon-related genes during follow-up, suggesting that RSV downregulated interferon-related responses (Mejias et al., 2013). The interferon pathway was also upregulated in blood transcriptomes of individuals with ARI that was caused by presence of virus (Zhai et al., 2015).

Given the distinct presence of bacterial, viral, or both (codetection) transcripts in NPAs, we sought to determine whether a specific immune signature could be identified in each group. We thus correlated the expression of immune genes with microbial loads and performed modularity analysis, which enabled us to identify a modular community of genes that were expressed in response to bacterial presence, viral presence, or both (codetection). The bacterial modular community contained 156 genes, associated with a variety of functions, including chemokines, transcription factors, cytokines, and genes related to TNF. A C4b-binding protein (C4BPA), that inhibits the classical pathway of complement activation and subsequent opsonization followed by lysis, was correlated with bacterial load, as well as bacterial plus viral (codetection) load. The C4BPA binds to several bacterial pathogens, including *H. influenzae* (Hallstrom et al., 2007) and *M. catarrhalis* (Nordstrom et al., 2004), enhancing bacterial adhesion, for example. Among the 29 genes positively correlated with viral load, two (CXCL11 and CXCL10) are interferon-regulated chemokines. Together with CXCL9, CXCL10, and CXCL11 bind to the same CXCR3 receptor and preferentially attract activated T- and NK- cells. Recent evidence points toward non-redundant roles for three IFN-induced CXCR3 ligands *in vivo*, for example, CXCL10 during viral infections and CXCL11 during allergic reactions (reviewed in Metzemaekers et al., 2018). This finding is in line with our results in children with ARI. Moreover, in comparison to the 5 genes observed in the codetected modular community, the 28 genes present in the viral modular community displayed significantly higher evolutionary antiviral scores (Supplementary Figure 4). These results were based upon genome-wide cross-species comparison of type I IFN-induced antiviral response (Shaw et al., 2017), underscoring the biological link between the viral gene module and viral load in ARI.

We also compared the immune response of NPAs that have clearly detectable microbial load (bacterial, viral, or codetection) with those that are classified as indeterminate. The comparison between viral or codetection NPAs against indeterminate NPAs showed that IFNL3 was uniquely shared between the two clinical groups, and significantly correlated with viral load. Type III IFN induces a variety of ISGs, interfering with viral replication (Sadler and Williams, 2008), and it seems to be the major source of IFN in airway epithelial cells (Khaitov et al., 2009). The IFNL3 is expressed in response to RSV (Spann et al., 2004) and IV (Durbin et al., 2013), both present in NPA samples. We also performed additional analysis of ten post-ARI samples from the larger cohort of 576 children, collected at 2–4 weeks after recruitment and diagnosis. These samples were subjected to nCounter analysis and compared with all the 53 samples that had detectable pathogens (bacterial, viral, and codetection groups). Total viral load decreased from 990 to 24 normalized counts (almost forty-fold) in cured patients after 2-4 weeks of follow-up (data not shown). This result was not statistically significant (*p* = 0.30), due to low numbers and a high coefficient of variation (>130%). On the other hand, bacterial loads remained equally high (>10,000 normalized counts) in both cured (post-ARI) samples and samples from ARI during the acute phase. We also found a roughly fivefold decrease in *IFNL3* transcripts, from a median of 32.2 (IQR 5.3–114.8) to 6.7 (IQR 2.7–57.9) normalized counts (*p* = 0.22). With regard to *IFNL3* transcripts, although decreased, they still correlated positively with both viral (*r* = 0.62, *p* = 0.06) and bacterial load (*r* = 0.62, *p* = 0.067) in post-ARI samples. No such trend was observed for type I IFN transcripts, either *IFNA2* or *IFNB1* (*p* > 0.2 for both bacterial and viral load). Therefore, these results emphasize the hypothesis that type III IFN might play a protective role in the ongoing antiviral and, possibly, antiviral immune response (and possibly the antibacterial immune response) in the nasal mucosa, even outside the symptomatic phase of ARI in pediatric patients. Because of the small size and short follow-up (2–4 weeks after ARI diagnosis), these findings remain to be confirmed in larger cohorts with prolonged clinical and microbiological followup.

The comparison between viral or codetection NPAs against indeterminate NPAs showed that IFNL3 was modulated. On the contrary, when the above comparisons were made against NPAs from healthy individuals, IFNL3 was not observed. This suggests that IFNL3 represents a marker of ARI. Type III IFN induces a variety of ISGs that interfere with viral replication (Sadler and Williams, 2008). It seems to be the major source of IFN in airway epithelial cells (Khaitov et al., 2009) and IFNL3 is expressed in response to RSV (Spann et al., 2004) and IV (Durbin et al., 2013), both of which are present in NPA samples.

Moreover, MAF was modulated in bacterial, viral, and codetection in comparison with indeterminate NPAs. The protein c-MAF, encoded by the *MAF gene*, is a DNA-binding, leucine zipper-containing transcription factor that, depending on the binding site and binding partner, can be a transcriptional activator or repressor. It plays a role in the regulation of several cellular processes. The protein c-MAF is one of the transcription factors signaling for the development of T follicular helper T cells (Tfh) from naïve CD4+ T cells (Andris et al., 2017). This development requires sequential steps involving cytokine, surface receptor, and transcription factor signaling. The Tfh cells are critical for the humoral response, that is, activation of B cells, class switching, and germinal center formation (Jogdand et al., 2016). This result suggests that MAF may be a key regulator in the immune response of ARI, regardless of the type of microbial presence.

Finally, when we compared the immune genes modulated in the comparisons against NPAs from healthy individuals vs. those against NPAs classified as “indeterminate” (Figure 7), we identified three categories of genes: immune genes specific to the presence of a microbial load, immune genes specific to the presence of clinical symptoms, and a group of genes at the intersection of these two settings. We propose that the latter group may act as markers of the immune response at the nasopharyngeal interface in children with ARI. Importantly, MARCO is a scavenger receptor expressed in tissue-resident macrophages that recognizes various bacterial pathogens, and MARCO, for example, can enhance adenovirus infection (Maler et al., 2017). On the other hand, CSFR1 and CSF2RB are the receptors for the major monocyte/macrophage growth factors M-CSF and GM-CSF, respectively. Thus, monocytes/macrophages may provide a bridge between innate and adaptive immunity in the nasal mucosa during ARI, clustering with lymphocyte activation markers IKAROS (encoded by IKZF1) and JAK3 (a central kinase in lymphokine signaling).

On the other hand, the presence of myeloid markers (CSFR1, CSFR2B, MARCO, and IRF5), costimulatory molecules (TNFSF8), and coinhibitory molecules (“SLAM Accessory Protein” SAP/SH2D1A) observed in bacterial NPAs, paralleled by the presence of coinhibitory molecules CD39 (encoded by the ENTPD1 gene, preferentially expressed in Tregs), exhaustion marker PD-L2 (encoded by the PDCD1LG2 gene), and inhibitory Fc receptor FCGR2B (containing an ITIM motif) was known to dampen the local immune response. As our study had a cross-sectional design, we cannot infer temporality or causality for “deleterious” or “protective” immune signatures, but the robust correlation between both bacterial and viral load, and IFNL3/IL28B, together with its documented antiviral and immunomodulatory roles (reviewed in Andreakos et al., 2017), suggests a pivotal role for type III IFN in maintaining tissue homeostasis during ARI.

Due to the cross-sectional design of the study and ethical limitations, we were unable to sample healthy age-matched infants. This is an inherent limitation of the study for the initial exploratory analysis, comparing NPAs from children with ARI vs. NPAs from healthy adults. However, our study was sufficiently powered to reveal distinct immune signatures linked to specific viral vs. bacterial pathogens in symptomatic children, in the second part of the study. Thus, we identified unique *in situ* immune signatures for bacterial and viral presence NPAs, which can help elucidate factors contributing to ARI in pediatric patients, leading to identification of candidate molecules for future immunotherapeutic or immunoprophylactic interventions.

## Data Availability Statement

All analyzed datasets for this study are included in the manuscript and the Supplementary Files.

## Author Contributions

KF and JVW designed the experiments. KF performed the experiments and analyses. CN-C, MB, and JO collected the samples. AB, TD, RK, JVW, and HN contributed to analysis. KF, CN-C, JVW, BA, and CO wrote the manuscript. All authors read and approved the final manuscript.

## Conflict of Interest Statement

The authors declare that the research was conducted in the absence of any commercial or financial relationships that could be construed as a potential conflict of interest.

## Footnotes

1 http://www.nanostring.com/products/gene_expression_panels
