# Supplementary material for: In situ Immune Signatures and Microbial Load at the Nasopharyngeal Interface in Children With Acute Respiratory Infection
Source: Front Microbiol. 2018 Nov 9;9:2475. doi: 10.3389/fmicb.2018.02475 (PMC6238668; doi:10.3389/fmicb.2018.02475)
Supplement: Supplementary file 1 [file Presentation_1.PDF]

In situ immune signatures of nasopharyngeal microbial load in children with Acute Respiratory Infection

Kiyoshi F. Fukutani[a], Cristiana M. Nascimento-Carvalho[b,c], Maiara L. Bouzas[b], Juliana R. Oliveira[b], Aldina Barral[a,b], Tim Dierckx[d], Ricardo Khouri[a,b], Helder I. Nakaya[e], Bruno B. Andrade[a,f], Johan Van Weyenbergh[d,#], Camila I. de Oliveira[a,b,#,*]

Supplemental Material

Supplemental Figure 1

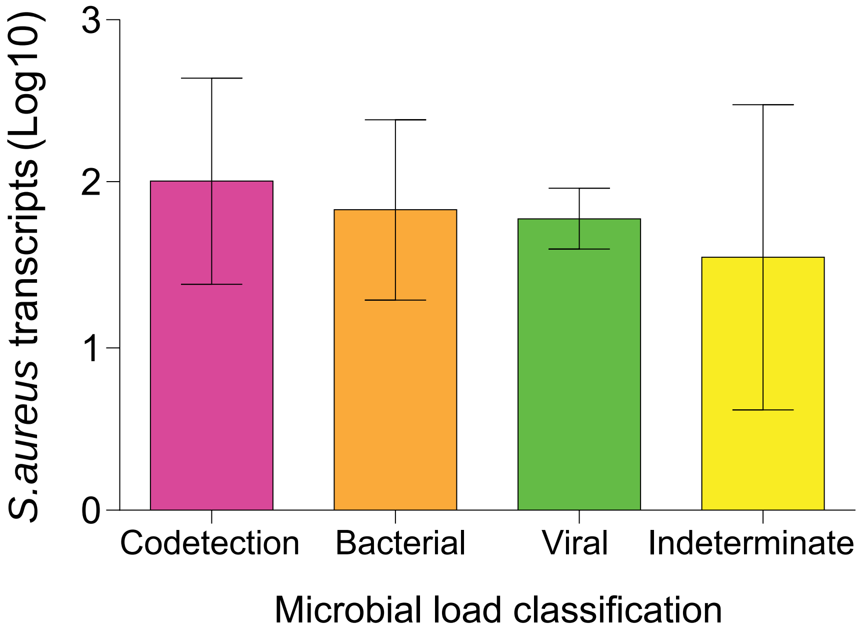

Supplemental Figure 1. Number of transcripts for *Staphylococcus aureus* in nasopharyngeal aspirates (NPAs) from children with ARI. NPAs were submitted to transcriptomic analysis targeting *S. aureus*. Bars (mean plus standard deviation) depict number of the transcripts (Log10) for each NPAs, according to the microbial load classification.

Supplemental Figure 2

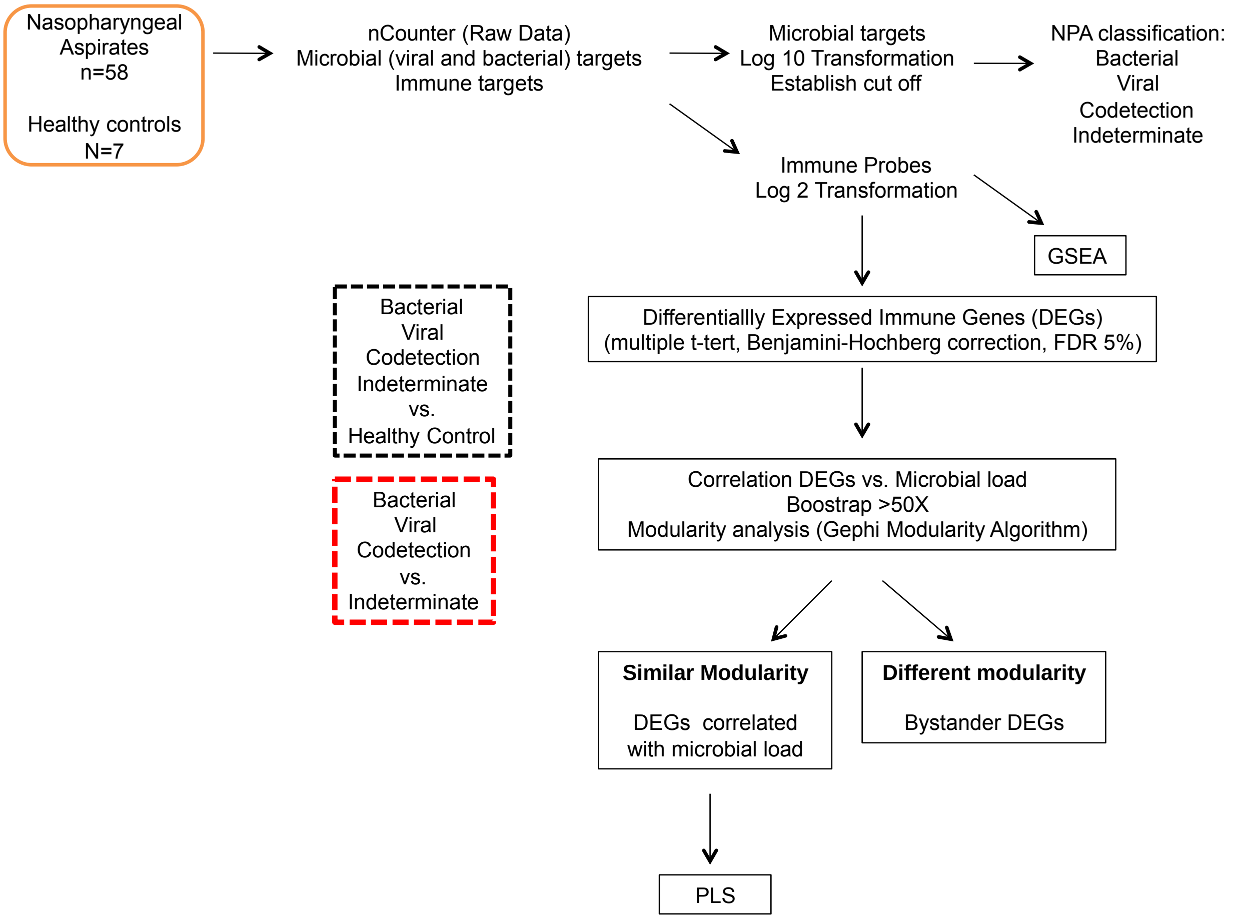

Supplemental Figure – Data analysis pipeline to determine microbial counts in in nasopharyngeal aspirates (NPAs) from children with ARI.

Supplemental Figure 3

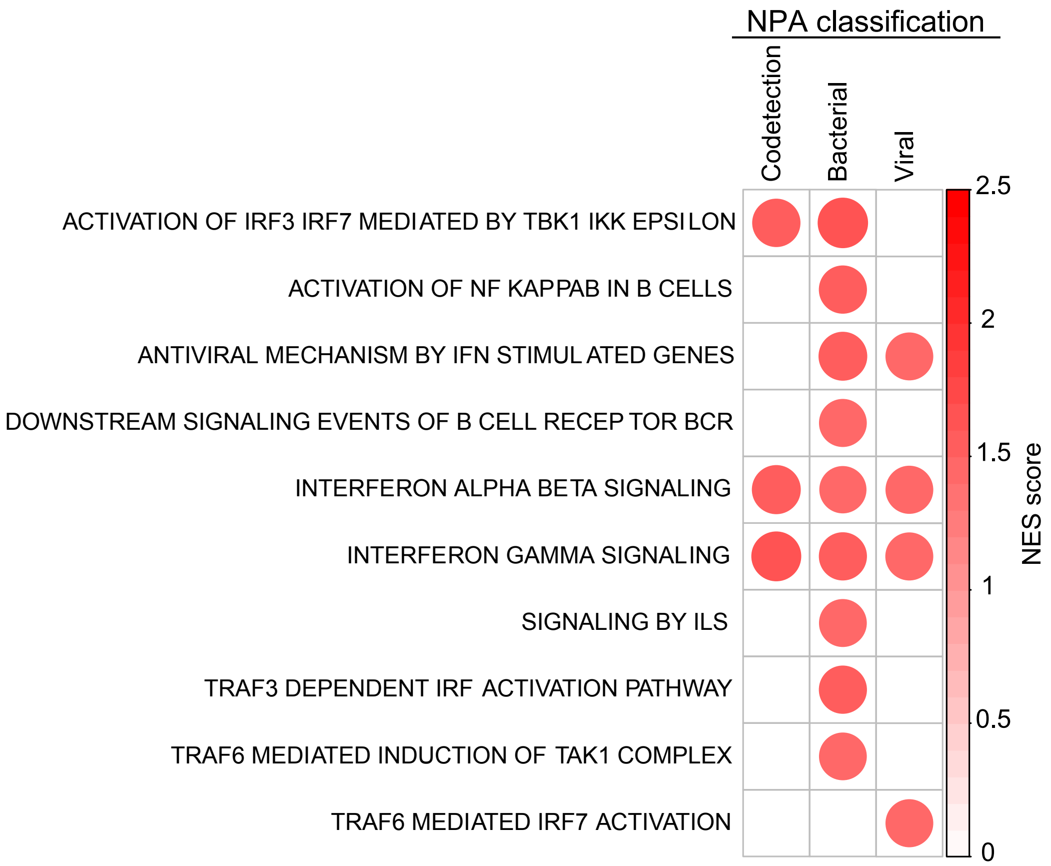

Supplemental Figure 3. Pathway enrichment analysis of immune genes expressed in nasopharyngeal aspirates of children with ARI. GSEA (Gene Set Enrichment Analysis) was performed to identify gene sets enriched for significantly modulated genes in NPAs classified according to the microbial load. Size of circles represents the number of genes in each pathway. Color intensity of circles represents the Normalized Enrichment Score (NES). Red indicates up-regulation.

Supplemental Figure 4

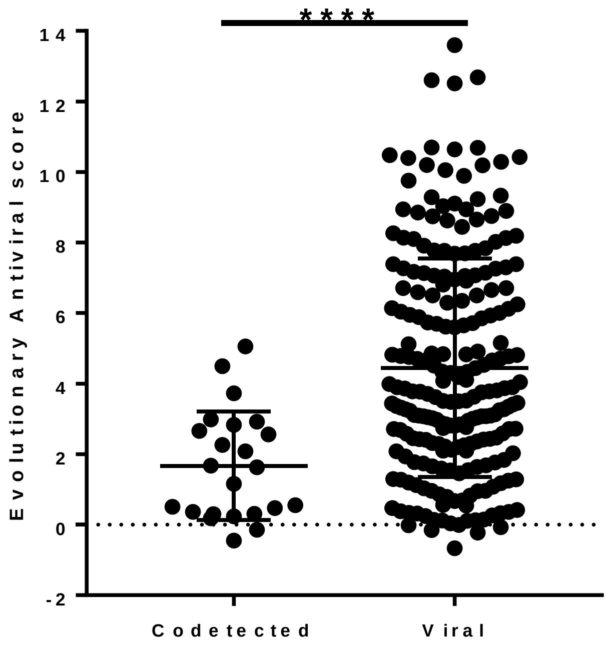

Supplemental Figure 4. The viral modular community represents evolutionary conserved antiviral genes. The publicly available database http://isg.data.cvr.ac.uk/ catalogues the interferomes of ten different species (human, mammals, chicken). Evolutionary antiviral scores for orthologous genes were defined by median fold-change of type I IFN up- or down-regulation during an antiviral state in cells from any of ten different species, using a string genome-wide correction (FDR <0.05). Median evolutionary antiviral score was significantly higher for the viral gene module, as compared to the codetection module (Mann-Whitney test, $p<0.0001$).
